# A Son of Life, and Other Poems

**Published:** 1920-10-30

**Authors:** 


### A Song of Life, and other Poems.

Since we have had the pleasure of commending Mr. Davies's previous volumes for their freshness and simplicity, our readers will be ready to welcome the latest verse of one of the two or three unquestionably genuine poets now writing. A quiet life with nature is still his choice, and to those who say that he "has no depth" because

> He writes of birds, of staring cows, and sheep, he replies wittily that he is not more silent than Time or Death, with whom some men ask him to be busy. His great gift is to see familiar things, as if no one else had ever seen them, and to describe them in words which have never been used before. A meteor to him is a tear running down the face of the sky, and a September landscape is vividly seen as follows:

> A summer's morning that has but one voice;
> Five hundred stooks, like golden lovers, lean
> Their heads together, in their quiet way,
> And but one bird sings, of a number seen.

We have no space to quote more from the thirty-six short lyrics which precede the poem which gives its title to the book. One, entitled "You Interfering Ladies," carries a warning to indiscreet health visitors and expresses the latter's activities from the point of view of the poor. The entire book brings a breath of country air along with it, and the sister and convalescent with any taste for letters will find here exactly what they need. Nothing changes Mr. Davies. He sings like the birds, and because he is really like them we never weary of listening.

* *A Song of Life, and other Poems.* By William H. Davies. (London: A. C. Fifield, 13 Clifford's Inn, E.C. 4. Price 5s. net.)

